# Maximum linkage space-time permutation scan statistics for disease outbreak detection

**DOI:** 10.1186/1476-072X-13-20

**Published:** 2014-06-10

**Authors:** Marcelo A Costa, Martin Kulldorff

**Affiliations:** 1 Department of Production Engineering, Universidade Federal de Minas Gerais, Belo Horizonte, Brazil; 2 Department of Population Medicine, Harvard Medical School and Harvard Pilgrim Health Care Institute, Boston, USA

**Keywords:** Spatial scan statistics, Space-time permutation, Sequential Monte Carlo

## Abstract

**Background:**

In disease surveillance, the prospective space-time permutation scan statistic is commonly used for the early detection of disease outbreaks. The scanning window that defines potential clusters of diseases is cylindrical in shape, which does not allow incorporating into the cluster shape potential factors that can contribute to the spread of the disease, such as information about roads, landscape, among others. Furthermore, the cylinder scanning window assumes that the spatial extent of the cluster does not change in time. Alternatively, a dynamic space-time cluster may indicate the potential spread of the disease through time. For instance, the cluster may decrease over time indicating that the spread of the disease is vanishing.

**Methods:**

This paper proposes two irregularly shaped space-time permutation scan statistics. The cluster geometry is dynamically created using a graph structure. The graph can be created to include nearest-neighbor structures, geographical adjacency information or any relevant prior information regarding the contagious behavior of the event under surveillance.

**Results:**

The new methods are illustrated using influenza cases in three New England states, and compared with the cylindrical version. A simulation study is provided to investigate some properties of the proposed arbitrary cluster detection techniques.

**Conclusion:**

We have successfully developed two new space-time permutation scan statistics methods with irregular shapes and improved computational performance. The results demonstrate the potential of these methods to quickly detect disease outbreaks with irregular geometries. Future work aims at performing intensive simulation studies to evaluate the proposed methods using different scenarios, number of cases, and graph structures.

## Background

The prospective space-time permutation scan statistic [1] is a widely-used method for early disease outbreak detection surveillance systems [2,3]. As opposed to the retrospective evaluation of disease cluster [4], its aim is to quickly detect an emerging infectious disease outbreak in a prospective manner through daily or weekly data feeds and repeated statistical analyses. The statistic requires no information regarding the underlying population at risk. Rather, it compared the recent geographical distribution of cases in comparison to historical counts, searching for space-time interaction clusters, and adjusting for any purely spatial or purely temporal variation in the counts. A cylinder structure with variable radius and height is used to scan the space-time region in order to select the candidate cluster with the maximum likelihood ratio statistic. The base of the cylinder represents the spatial component, and its height defines the time interval at which the cluster is allegedly located. A Monte Carlo simulation is performed to estimate the p-value using a data permutation procedure under the null hypothesis of no cluster.

A key component of the spatial scan statistic is the geometry of the scanning window. Originally, squares or rectangular windows were the primary shapes proposed [5]. Subsequent works applied circles, ellipses [6] and triangles [7,8]. More recently, spatial scan statistics have been applied using non-parametrical windows that take very irregular shapes [9-16].

Extensions of the irregular shaped scan statistic to the space-time setting have been a challenge. To augment the flexibility of the search procedure to handle an irregular shaped scanning window in three dimensions, requires more complex algorithms and more elaborated data structures. Consequently, more computing effort is needed. In disease surveillance, it is important to detect disease outbreaks as soon as the data are available, and therefore, computationally efficient methods must be designed. Takahashi et al. [17] proposed one such method using a nonparametric definition of the spatial extent of the cluster. It relies on a cylinder scanning window in which the base of the cylinder also assumes non-circular shapes. In brief, if the base is composed of a maximum of *K* regions and the center of the base is region *i* then irregular shapes, which must include region *i*, are created using the combinations of the remaining *K* - 1 regions. The method does not consider cluster candidates in which the regions are not connected, nor clusters that change its geographical size and shape over time.

In this paper, we propose an irregular shaped space-time permutation scan statistic that allow for two types of flexibility in the cluster shape. In the first proposal the cluster can be irregular only in space, which means that the spatial extent of the cluster is the same during the whole time period of the cluster. In the second proposal the cluster can be flexible in both space and time. This latter type is more informative, since it tries to incorporate the differential spatial extent of the cluster over time.

The two proposed methods were evaluated by mimicking a prospective surveillance system for influenza events during 2005, using daily data from Harvard Pilgrim Health Care, and using a simulation study. The methods were compared to the cylindrical space-time permutation scan statistic. Results show that all methods provided good outbreak detection. The irregular shaped versions provide more details about the spatial and temporal extent of the outbreak. The cylindrical version is much faster computationally. This is so because the computational effort to create cylindrical shapes are much smaller than creating irregular candidates. Due to the computational complexity, the standard Monte Carlo inference represents a major burden in the time required to perform the disease surveillance with irregular shapes. Alternatively, we apply sequential Monte Carlo which provides an early stop of the Monte Carlo simulations if there is enough evidence from the simulated values that the null hypothesis is rejected. Consequently, it reduces the computational burden in the irregular cluster detection.

## Results and discussion

### Case study: influenza outbreak detection

**Figure 1:**
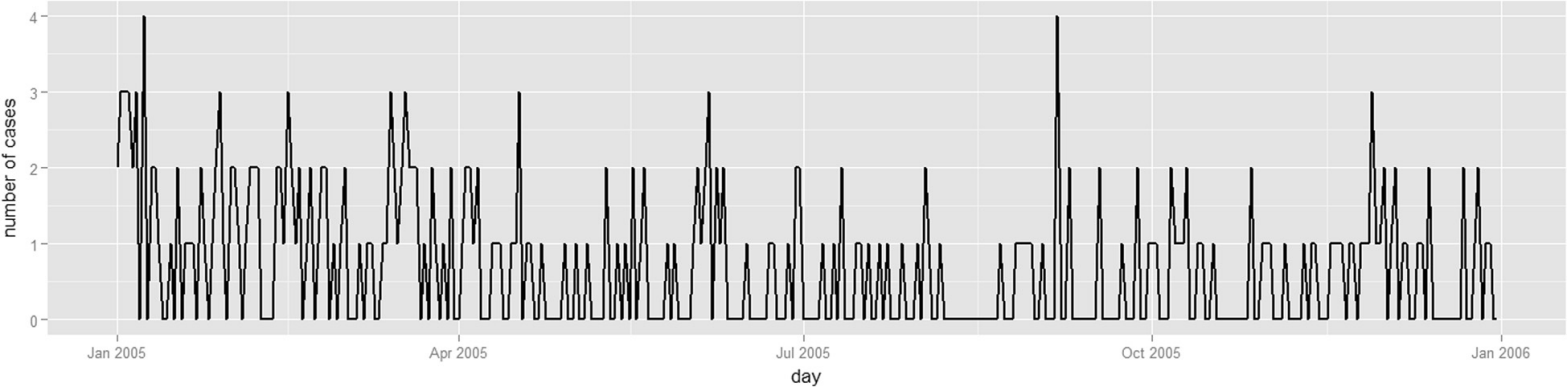
Daily incidence of Influenza in patients age 13 and over from hospital emergency departments during 2005.

**Figure 2:**
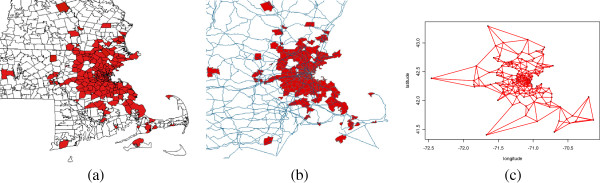
Final graph structure (c) generated using geographic adjacency information (a) and main transportation routes (b).

We evaluated the new methods by mimicking a daily prospective analysis of historical cases of influenza in patients aged 13 and over who (i) went to a hospital emergency department in or around Boston, USA, and who (ii) had health insurance coverage from Harvard Pilgrim Health Care. Data from January 1, 2005, to December 31, 2005, were used with a total of 249 reported cases in 206 counties in Massachusetts, New Hampshire and Rhode Island. The data was provided by the Harvard Pilgrim Health Care Insurance Company. Figure 1 shows the daily number of visits.Figure 2a shows locations by ZIP code. Some of the areas in which cases were reported are scattered across the geographic region depicted. Consequently, if the graph structure is built just based on geographic adjacency, then some areas will not be connected in the graph. In order to create an alternative graph structure in which each area is connected to at least two other areas, the adjacency structure was partially merged with the main transportation routes (Figure 2b) and the two nearest-neighbors. The final graph structure is shown in Figure 2c.

In disease surveillance and outbreak detection systems, graph structures are extremely useful, since they allow to incorporate a variety of information in the same structure. In this application, further information such as data on trains, buses or subways could have been used. It is of special interest if the graph information is strongly correlated to the event under surveillance. For example, main roads were used as an upgrade to the spatial graph due to the fact that automobiles are the most common transportation system in the studied region.

For the monitoring system, a historical window of one year was used to estimate the marginal and expected number of cases in the candidate clusters under the null. The maximum temporal window length was set as seven days. The maximum geographical size of the cluster was chosen to be 50% of the total number of cases for the cylindrical method, and 100 zip-code areas for the two irregular *mlink* methods. The number of Monte Carlo simulations was set to 9,999 for the cylindrical scan statistic. The irregular shape procedures used sequential MC parameters *h = 50* and *n = 999*. It is worth noting that the irregular geometry search procedures do not necessarily consider all possible cylindrical subsets as candidates. Therefore, a signal detected by the cylinder approach is not necessarily detected by the irregular *mlink* methods, and vice versa.

Table 1 shows the results for the surveillance system for Influenza during 2005. Overall, 41 days presented signals with recurrence interval greater than 100 days. If we account time overlap among the signal then 13 distinct signals were found. We only evaluated detected clusters with more than one case, even though clusters with only one case may present a high Recurrence Interval. Therefore, Table 1 shows 23 days with recurrence intervals greater than 100 days and 9 distinct overlapping signals.

**Table 1:** **Mimicking real-time analysis of emergency department visits due to influenza for individuals age 13 and over using cylindrical and irregular shaped space-time permutation scan statistics (*mlink-space *** **and *mlink space-time*)**

**Date**	**Method**	**Number of days in the signal**	**Number of ZIPs in the signal**	**Observed cases**	**Expected cases**	**Relative risk**	**pvalue**	**Recurrence interval**
24-Jan-05	Mlink space-time	2	2	2	0.07	27.1	0.0630	16
	Mlink space	2	29	3	0.25	12.1	0.1526	7
	Cylindrical	4	16	3	0.12	24.2	0.0049	204
25-Jan-05	Mlink space-time	3	3	2	0.07	27.1	0.0320	31
	Mlink space	3	29	3	0.25	12.1	0.0930	11
	Cylindrical	5	16	3	0.12	24.2	0.0049	204
26-Jan-05	Mlink space-time	4	6	2	0.07	26.9	0.0746	13
	Mlink space	4	16	3	0.23	13.0	0.0739	14
	Cylindrical	6	16	4	0.30	13.4	0.0051	196
28-Jan-05	Mlink space-time	4	5	2	0.12	16.1	0.3049	3
	Mlink space	6	5	3	0.17	17.2	0.0260	38
	Cylindrical	7	34	5	0.45	11.0	0.0016	625
29-Jan-05	Mlink space-time	5	11	2	0.17	11.9	0.5102	2
	Mlink space	7	8	3	0.20	14.8	0.0726	14
	Cylindrical	7	34	5	0.54	9.3	0.0073	137
15-May-05	Mlink space-time	3	4	2	0.04	45.5	0.0040	250
	Mlink space	6	30	3	0.43	7.0	0.3623	3
	Cylindrical	7	6	2	0.06	31.4	0.0416	24
17-May-05	Mlink space-time	3	3	1	0.02	48.0	0.4132	2
	Mlink space	3	5	2	0.07	26.7	0.0288	35
	Cylindrical	7	15	3	0.14	21.3	0.0019	526
18-May-05	Mlink space-time	4	8	2	0.07	26.7	0.0210	48
	Mlink space	4	5	2	0.07	26.7	0.0551	18
	Cylindrical	7	15	3	0.14	21.3	0.0019	526
19-May-05	Mlink space-time	5	9	2	0.07	26.7	0.0430	23
	Mlink space	5	5	2	0.07	26.7	0.0752	13
	Cylindrical	7	15	3	0.14	21.3	0.0064	156
26-Jul-05	Mlink space-time	6	9	2	0.09	21.7	0.0050	200
	Mlink space	6	2	2	0.12	16.7	0.0602	17
	Cylindrical	7	3	2	0.12	16.7	0.1220	8
2-Sep-05	Mlink space-time	6	5	3	0.15	20.4	0.0080	125
	Mlink space	6	2	3	0.19	15.6	0.0666	15
	Cylindrical	6	3	3	0.21	14.0	0.0888	11
3-Sep-05	Mlink space-time	7	5	3	0.15	20.4	0.0030	333
	Mlink space	7	2	3	0.19	15.6	0.0452	22
	Cylindrical	7	3	3	0.21	14.0	0.0735	14
27-Sep-05	Mlink space-time	5	8	2	0.08	25.4	0.0090	111
	Mlink space	5	7	2	0.08	25.4	0.0164	61
	Cylindrical	7	12	2	0.20	9.8	0.4350	2
28-Sep-05	Mlink space-time	6	13	2	0.08	25.4	0.0050	200
	Mlink space	6	7	2	0.08	25.4	0.0148	67
	Cylindrical	7	12	2	0.20	9.8	0.4600	2
27-Oct-05	Mlink space-time	1	2	2	0.11	17.5	0.0010	1000
	Mlink space	1	2	2	0.11	17.5	0.0010	1000
	Cylindrical	7	18	2	0.11	17.5	0.0581	17
28-Oct-05	Mlink space-time	2	2	2	0.11	17.5	0.0010	1000
	Mlink space	2	2	2	0.11	17.5	0.0010	1000
	Cylindrical	7	18	2	0.11	17.5	0.0581	17
29-Oct-05	Mlink space-time	3	2	2	0.11	17.5	0.0010	1000
	Mlink space	3	2	2	0.11	17.5	0.0010	1000
	Cylindrical	7	18	2	0.11	17.5	0.0581	17
30-Oct-05	Mlink space-time	4	2	3	0.14	21.8	0.0010	1000
	Mlink space	4	2	3	0.14	21.8	0.0010	1000
	Cylindrical	7	1	3	0.14	21.8	0.0007	1429
31-Oct-05	Mlink space-time	5	1	3	0.14	21.8	0.0020	500
	Mlink space	5	2	3	0.14	21.8	0.0010	1000
	Cylindrical	7	11	4	0.26	15.4	0.0007	1429
1-Nov-05	Mlink space-time	6	2	3	0.14	21.8	0.0070	143
	Mlink space	6	2	3	0.14	21.8	0.0080	125
	Cylindrical	7	11	4	0.29	13.9	0.0026	385
2-Nov-05	Mlink space-time	7	2	3	0.14	21.8	0.0060	167
	Mlink space	7	2	3	0.14	21.8	0.0050	200
	Cylindrical	7	11	4	0.29	13.9	0.0026	385
19-Nov-05	Mlink space-time	5	10	2	0.13	15.8	0.2137	5
	Mlink space	7	11	4	0.32	12.6	0.0070	143
	Cylindrical	7	8	3	0.21	14.4	0.0643	16
19-Dec-05	Mlink space-time	7	4	1	0.06	17.0	0.0020	500
	Mlink space	7	2	1	0.06	17.0	0.2542	4
	Cylindrical	7	54	2	0.18	11.3	0.1820	5

For *mlink space-time*, the number of zip-code areas in the signal is the number of distinct ZIP codes over the temporal length of the cluster, regardless of their temporal position. Overlapping signals are grouped in the table.

From January 24, 2005, to January 29, 2005, the cylinder method detected a sequence of overlapping signals, as can be seen by the progressive increase of their temporal length. The arbitrary shape methods did not detect any strong signals during this period.

On May 15, 2005 the *mlink space-time* detected a signal which was later detected by the cylindrical method on May, 17, 18, and 19, 2005, respectively. The *mlink space-time* method also detected a signal on July 26 with 2 cases and Recurrence Interval of 200 days.

**Figure 3:**
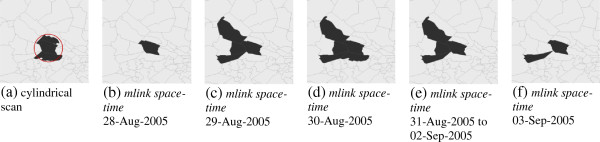
**Comparison of the clusters detected with the cylindrical and *mlink space-time *permutation scan statistics on 03-Sep-2005.** The arbitrary cluster starts with one ZIP code, and it gradually increases until it reaches five ZIP codes. Then the cluster begins to vanishing.

From September 2, 2005 to September 3, 2005, two overlapping signals were detected by the *mlink space-time*. Figure 3 shows the detected clusters using the cylindrical and the *mlink space-time* methods on September 3, 2005. The base of the cylindrical cluster is shown in Figure 3a whereas Figures 3b to 3f shows the temporal dynamics of the cluster found with *mlink space-time* in which the arbitrary cluster starts with one ZIP code, and it gradually increases until it reaches five ZIP codes. Then the cluster begins to vanishing on September 3, 2005.

Overlapping signals were also found by the arbitrary shape methods from September 27 to September 28, 2005. The *mlink space-time method* detected both signals.

From October 27, 2005 to October 31, 2005, a new sequence of overlapping signals was simultaneously detected by all methods. In particular, the *mlink space-time* and *mlink space* methods were the first to detect the signal. From November 1, 2005 to November 2, 2005, all methods detected the same signal.

Finally, one signal was detected by *mlink-space* on November 19, 2005, and one signal was detected by *mlink space-time* on December 19, 2005.

**Figure 4:**
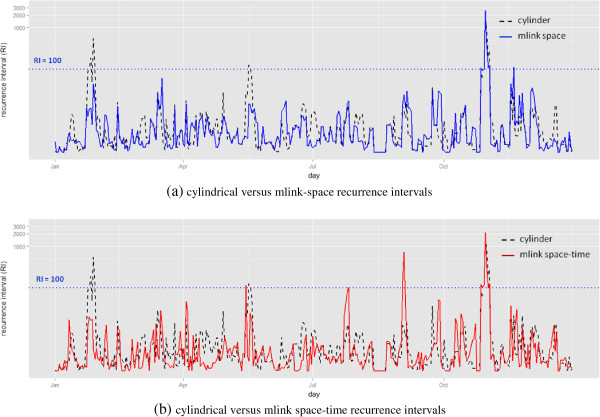
**Comparison of the Recurrence Intervals (1/p-value) for signals detected in 2005 for methods: cylindrical scan statistic versus *mlink-space *** **(a), and cylindrical scan statistic versus *mlink space-time *** **(b).**

Figure 4 compares the patterns of the signals detected by the cylindrical, the *mlink-space*, and the *mlink space-time* permutation scan statistics. In general, the shapes of the curves are different, and all methods share some regions in which the same signal is detected.

### Simulation study

In order to investigate some properties of the proposed arbitrary cluster detection techniques, we designed the following simulation study. We first evaluated the number of cases within the period of one year from December 31^st^ 2004 to December 30^th^, 2005. The total number of cases in the period is 254 with a daily average of 0.69 cases/day. Second, under the null hypothesis of no spatial temporal clusters we randomly sampled with replacement 254 ZIP codes which showed at least one case in the time period. The probability of the sampling was proportional to the number of cases in each ZIP code.

Third, we also sampled with replacement 254 dates which were also selected from the observed dates in the time period. The probability of sampling was also proportional to the number of cases in each day. Then, we matched the sampled ZIP codes with the sampled dates. By doing so, the marginal distributions of the observed and the simulated data sets are the same.

**Figure 5:**
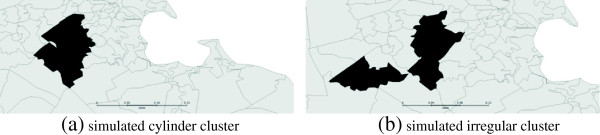
**Spatial geometries of simulated clusters.** The compact cluster **(a)** has 6 ZIP codes and the irregular cluster **(b)** has 5 ZIP codes.

We simulated separately one irregular dispersed cluster and one compact cluster. The spatial geometries of the clusters are shown in Figure 5. The irregular cluster has an irregular base as shown in Figure 5b. The geographical locations of the simulated clusters are close to the detected clusters in the case study data set. The time period of the clusters is from October 6^th^ 2010 to October 12^th^, or seven days. The irregular cluster has 5 ZIP codes and the compact cluster has 6 ZIP codes.

For the irregular and compact clusters the number of cases in the clusters was generated from a Poisson distribution with the mean parameter of 4.83, or 7 times the daily average of cases in the simulated period. Therefore, our simulations assume a relative risk of 7 in both the cylindrical and irregular clusters. The value of the relative risk equal to 7 was chosen based on the minimum observed relative risk found in our analysis using the real data set.

We generated 1,000 simulated data sets for the compact cluster and 1,000 simulated data sets for the irregular cluster. For each simulated data set we mimicked a surveillance system from October 1^st^ to 30^th^. Therefore, for each simulated data base we performed daily surveillance for 30 consecutive days using the cylindrical, *mlink-space*, and the *mlink space-time* permutation scan statistics.

**Figure 6:**
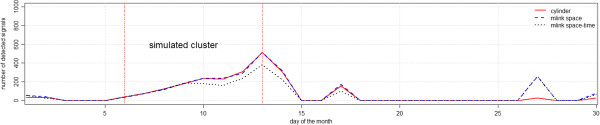
Number of detected signals with p-values smaller than 0.05 for irregular cluster simulations.

Figure 6 shows the number of simulations with p-values below 0.05 for the cylindrical, *mlink-space* and *mlink space-time* methods, for the irregular cluster simulations. The maximum number of detected signals was 513 which happened at the end of the time period of the simulated clusters. Both cylinder and *mlink-space* methods achieved similar results in detecting the cluster. All methods detected false signals after the 15^th^ day, nevertheless both *mlink-space* and *mlink space-time* methods detected more false signals than the cylinder method.

**Figure 7:**
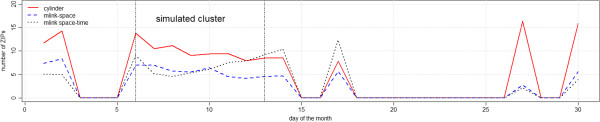
Average number of ZIP codes which were falsely detected, with p-value smaller than 0.05 for irregular cluster simulations.

Figure 7 shows the average number of ZIP codes which were falsely detected, that is, the average number of ZIP codes that do not belong to the real cluster, with p-values smaller than 0.05. Results show that the cylinder method detected, on average, larger clusters of false ZIP codes as compared to *mlink-space* and *mlink space-time* methods. The *mlink-space* clusters presented smaller number of false ZIP codes, on average. Just after the simulated cluster period, the *mlink space-time* detected a larger number of false ZIP codes, on average.

**Figure 8:**
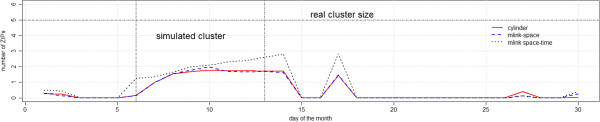
Average number of detected ZIP codes which belong to the real cluster, with p-value smaller than 0.05 for irregular cluster simulations.

Figure 8 shows the average number of ZIP codes which belong to the real cluster, with p-values smaller than 0.05. In general, both *mlink-space* and the cylinder methods detected the same number of ZIP codes, on average; whereas, the *mlink-space time* detected a larger number of ZIP codes from the real cluster.

**Figure 9:**
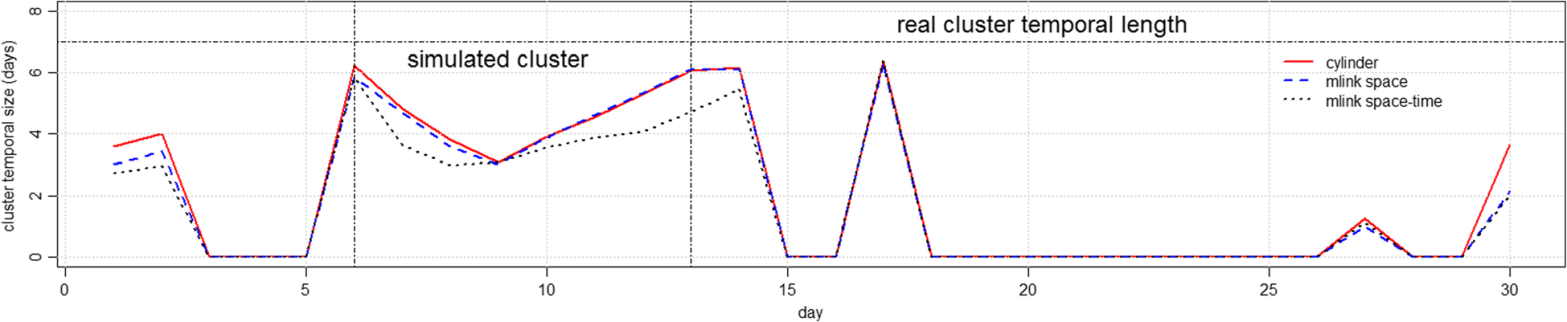
Average number of days in detected clusters with p-value smaller than 0.05 for irregular cluster simulations.

Figure 9 shows the average number of days, or the temporal length, of the detected clusters with p-values smaller than 0.05. In general, all methods detected clusters with temporal length that were smaller than the real temporal cluster length. Furthermore, false signals detected immediately after October 15^th^, on October 17^th^, overlap with the simulated clusters which indicate a latter detection of the true cluster.

**Figure 10:**
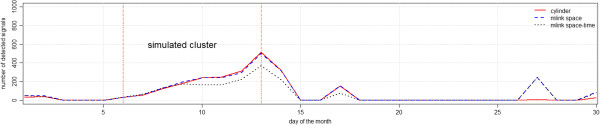
Number of detected signals with p-values smaller than 0.05 for compact cluster simulations.

**Figure 11:**
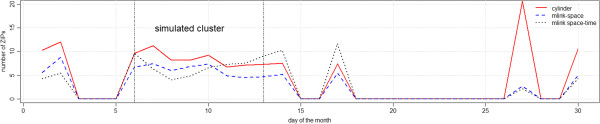
Number of ZIP codes which were falsely detected, with p-value smaller than 0.05 for compact cluster simulations.

**Figure 12:**
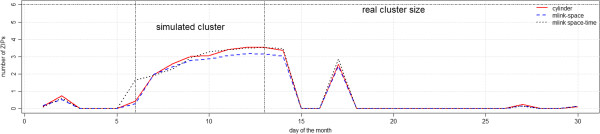
Average number of detected ZIP codes which belong to the real cluster, with p-value smaller than 0.05 for compact cluster simulations.

**Figure 13:**
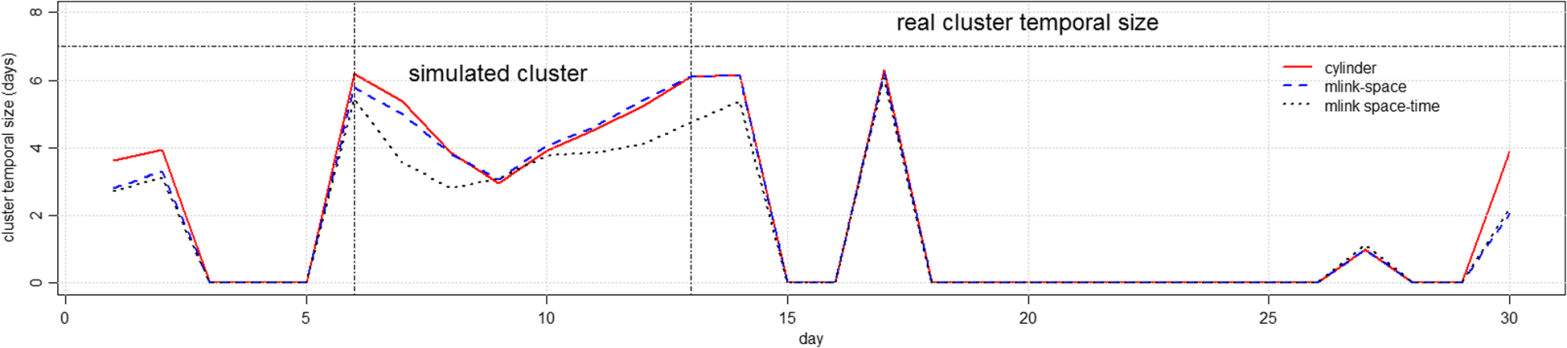
Average number of days in detected clusters with p-value smaller than 0.05 for compact cluster simulations.

Similar results were found for the simulated compact cluster as shown in Figures 10, 11, 12 and 13. In general, the cylindrical and *mlink-space* methods detected similar and higher number of signals than the *mlink space-time* method, as shown in Figure 10. The maximum number of detected signals was 514 which happened at the end of the time period of the simulated clusters. Figure 11 shows that the cylinder detected significant clusters with a larger number of false ZIP codes as compared to the *mlink space* and *mlink space-time*, on average. Just after the simulated cluster period, the *mlink space-time* detected a larger number of false ZIP codes, on average. Figure 12 shows that the cylinder and *mlink space-time* detected a higher number of ZIP codes from the real cluster, on average. The temporal length of the detected clusters for all methods were smaller than the real cluster, on average, as shown in Figure 13.

In general, our simulation study suggests that detected signals that last for one day may represent false signals. In addition, based on the fact that the simulated irregular and circular clusters have similar geographical sizes and locations as compared to some of the detected clusters in the original data base, our simulation study suggests that the *mlink space* and circular scan statistics share similar statistical power. Nevertheless, the simulations also suggest that the *mlink space* and *mlink space-time* scan statistics detects smaller clusters as compared to the circular scan statistic. Furthermore, clusters detected using *mlink* are closer to the real cluster size. In addition, if the real cluster is compact then *mlink-space* and cylinder methods detect more elements of the real cluster than *mlink space-time*, on average. Otherwise, if the real cluster is irregular then *mlink space-time* detects more elements of the real cluster than the cylinder and *mlink space* methods, on average.

## Conclusion

The performance of the prospective irregular shaped space-time permutation scan statistics, named *mlink-space* and *mlink space-time*, is promising. The proposed methods provide clusters detection in which the shape of the cluster can be irregular in space, and equally important, its spatial extent may shift over time. Another contribution of our proposed methods is its capacity to integrate a wide variety of information during the clustering process. The surveillance analysis can be adapted to specific aspects of the event under study such as social networks, transportation routes, and landscape information. By doing so, it is expected that an Euclidian based surveillance system and our proposed methods may detect different clusters, and both types may be used in parallel.

The simulation findings are similar to those previously found using the purely spatial *mlink*[16]. If the emphasis is on statistical power for cluster detection, the cylinder scan statistic is attractive. If the emphasis is on the accurate determination of the cluster size, shape and boundaries, maximum linkage space and space-time are more suitable choices. Therefore, irregular based methods, such as the *mlink* scan statistics, provide more accurate results regarding shapes and boundaries than circular or cylinder scan statistic.

The comparative conclusion of this paper on space-time clusters is similar to prior work on purely spatial clusters [16]. Takahashi et al. [17] is the only other paper we could find on irregular shaped space-time scan statistic. Briefly, all irregular scan statistics share the problem of computing burden which is crucial for epidemic surveillances. For instance, Takahashi et al. evaluate all possible irregular clusters within a predefined circle, but the circle cannot be too big for computational reasons. The two *mlink* methods evaluate fewer configurations of irregular clusters, not allowing for super irregular clusters. By doing so, they reduce the computing time and allows the detection of larger clusters.

As previously mentioned, our proposed methods do rely on intensive computing resources. Although our proposed methods were implemented using C\C++ language, the time required by the *mlink space-time* for a total of 999 Monte Carlo simulations is 480 seconds (or 8 minutes), on average, using a Intel i7 1.73GHz with 8GB. The cylinder approach is much faster and requires 24 seconds for a total of 999 Monte Carlo simulations. For *mlink*, the sequential Monte Carlo procedure reduces the number of simulations to 50, or 24 seconds, on average. The sequential Monte Carlo procedure can also be applied to the cylinder method which reduces the number of simulations to 50, or 1.2 seconds, on average. Nevertheless, if there is evidence that the null hypothesis is false during the sequential Monte Carlo, then the complete Monte Carlo simulation is required, and the sequential approach does not provide any time savings.

Alternatively, we evaluated the use of the Gumbel distribution as proposed by Abrams, Kulldorff and Kleinman [18] which fits the Gumbel distribution to a smaller set of simulated test statistics, say 499, and then estimate the p-value. However, the number of cases in our case study is very small, and as a consequence the distribution of the test statistic is mostly discrete. Thus, the Gumbel distribution did not fit properly to the distribution of the simulated test statistic.

While the focus on this paper has been on prospective surveillance systems for disease outbreak detection, the proposed methods can easily be adapted and applied to retrospective space-time analyses, where a historical data set is only analyzed once in order to detect space-time clusters during any time period covered by the data. Such analyses are used when there is less interest in the immediate public health need to detect emerging outbreaks, and more interest in understanding the general nature of the disease by studying its historical behavior.

For this paper, we generated the neighborhood structure using purely geographical information and main transportation routes. Other community characteristics such as socio-economic status (SES) could also be incorporated when defining close neighbors. For example, two poor communities may be closely connected even if they are not geographically adjacent, and combined geographical-SES based neighborhood structure could potentially help identifying health disparities generated clusters.

When doing actual surveillance to detect disease outbreaks, no statistical method can be expected to perform well unless the surveillance data is of high quality. For example, a reasonable proportion of the population in the surveillance area must be covered; a sufficient proportion of the covered individuals must be captured when they get sick; and it is critically import to be able to distinguish between one individual having two different health care encounters versus two different individuals having one encounter each [19].

In summary, we have successfully developed two new space-time permutation scan statistics methods with irregular shapes and improved computational performance, although the two proposed methods were not extensively evaluated for a wide variety of examples and in different scenarios. The methods were illustrated by mimicking a real daily influenza surveillance system. The results demonstrate the potential of these methods to quickly detect disease outbreaks with irregular geometries. Future work aims at performing intensive simulation studies to evaluate the proposed methods using different scenarios, number of cases, and graph structures.

## Methods

### The space-time permutation scan statistic

The prospective space-time permutation scan statistic [1] uses the historical number of cases instead of the population at risk to estimate the expected number of cases in geographical locations. Furthermore, the space time permutation scan statistic automatically adjusts for any purely spatial and purely temporal variation in the data, such as different health utilizations rates in different geographical locations, seasonal patterns of disease incidence and day-of-week variations.

The method uses a very large number of overlapping cylinders of different shapes and sizes to scan cluster candidates. The circular base of the cylinder represents space, and is typically centered at any of a large number of pre-defined centroids and has a continuously variable radii. The height of the cylinder represents time, which can be short or long. If the method is applied to retrospective studies, then the temporal component can have any start and end time. In this paper, we only consider the prospective version in which analyses are repeated on a daily or weekly basis as new data arrives. Thus, the end of the cylinder is always at the current time, while its start time varies corresponding to different possible start times of the disease outbreak.

To select a final cluster candidate, the method first estimates the expected number of cases inside each cylinder as the following. Let *c*_*zt*_ represents the observed number of cases in area *z* during time *t*. The total number of cases is

$$C=\underset{z}{\sum }\underset{t}{\sum }c_{\text{zt}}$$

The expected number of cases in a particular area *z* and time *t* is calculated based on the observed marginals,

$$\mu_{\text{zt}}=\frac{1}{C}\left(\underset{t}{\sum }c_{\text{zt}}\right)\left(\underset{t}{\sum }c_{\text{zt}}\right)$$

Next, the expected number of cases for a cylinder candidate *A* placed in a particular region and time interval is the sum of the expected number of cases for each temporal-area inside the cylinder,

$$\mu_{A}=\underset{\left(z,t\right)\in A}{\sum }\mu_{\text{zt}}$$

The test statistic is based on a Poisson approximation to the hypergeometric distribution of the observed number of cases in the cylinder *c*_*A*_. The Poisson generalized likelihood ratio, *κ* is

$$\kappa \left(A\right)={\left(\frac{c_{A}}{\mu_{A}}\right)}^{c_{A}}{\left(\frac{C-c_{A}}{C-\mu_{A}}\right)}^{\left(C-c_{A}\right)}$$

The cylinder candidate is the one with maximum likelihood ratio. To test the null hypothesis of space-time randomness, a Monte Carlo simulation [20] is performed. For each individual, the spatial location is held fixed, while a new random time is selected from the times observed over all individuals. This procedure does not change the spatial and temporal marginals. A large number of random permutations generate an empirical distribution under the null. The p-value is the proportion of simulated statistics higher than the observed one, that is, p = (R + 1)/(*n* + 1), where R is the rank of the observed test statistic, and *n* is the number of Monte Carlo simulations. This procedure maintains the alpha level exactly, as long as α⋅(*n* + 1) is an integer [21]. For recurrent analyses in a prospective surveillance system, we prefer to report the recurrence interval (RI) rather than the p-value. If analyses are done on a daily basis on daily data, RI = 1/p. If the null hypothesis is true, then the expected number of signals with RI > D is exactly one during any period of D days. For example, if the recurrence interval is 2 years and 6 months, then under the null, the expected number of signals of the same strength or greater, during a 2 ½ year period of daily analyses, is one.

### Maximum linkage space-time permutation scan statistic

**Figure 14:**
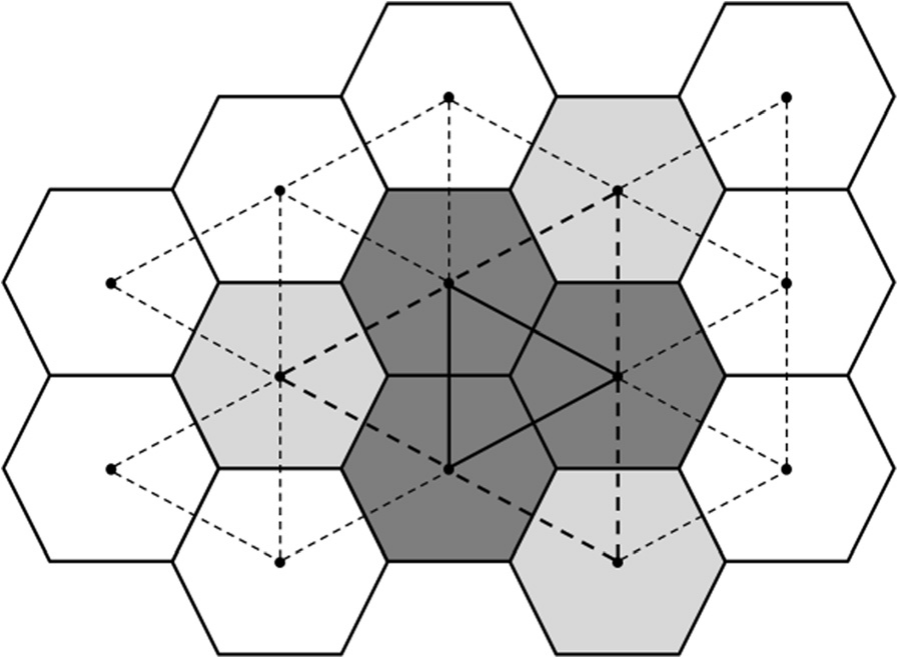
**The *mlink *method of the growth of a cluster candidate.** The graph structure was generated based on geographical adjacency. Dark areas represent the current cluster candidate, and gray areas are the areas most connected to the cluster. In the next step, the area that maximizes the most the likelihood will be incorporated. The process stops when the maximum cluster size is reached.

The maximum linkage irregular shaped space time permutation scan statistic is a generalization of the maximum linkage irregular shaped purely spatial scan statistic [16]. It is sometimes abbreviated as *mlink*. The *mlink* method creates cluster candidates based on adjacency graph structures. The method first considers each area as the initial cluster candidate and continuously evaluates the areas connected to the cluster by means of one or more edges. The criterion for selecting new areas is twofold. First, the method searches for the area that is the most connected to the current cluster or, equivalently, the area sharing the largest number of edges or connections to the current cluster. If two or more candidates share the same maximum number of connections, then the one capable of most increasing the likelihood ratio statistic is aggregated into the cluster; otherwise, the most connected area is automatically aggregated to the cluster. Furthermore, if the candidate areas do not contribute to increasing the cluster likelihood, then the one that least decreases the likelihood ratio statistic is selected. This process guarantees that at each step, the cluster incorporates one additional area. This continues until the cluster reaches a maximum size defined *a priori* by the user. The maximum number of connections or maximum linkage (*mlink*) criterion introduces a subtle but necessary and efficient constraint in the likelihood function. Duczmal and Assunção [9] and Assunção et al. [12] showed that if the increase of the likelihood statistic is the only criterion for selecting the next area to aggregate to the cluster, then detection power is compromised, and the detected clusters are usually oversized and oddly shaped, with many octopus-like tentacles going in different directions.Figure 14 illustrates the growing process of the spatial component of the cluster. In this example, information on geographical adjacency was used to design the graph structure. Dark areas represent the current cluster, and gray areas are potential candidates for aggregation. The candidate areas share exactly two connections with the cluster. The selection decision is based on the likelihood maximization or on the least decrease in likelihood.

The outline of the *mlink* algorithm is as follows.

#### The maximum linkage algorithm

**Step 1**. Start with one single area as the current cluster.

**Step 2**. Using the graph structure, sort neighbors according to their number of connections to the current cluster.

**Step 3**. Aggregate into the cluster the neighboring area with the maximum number of connections to the current cluster. That is, add the area outside the cluster that is connected to the most number of areas inside the cluster.

**Step 4**. If two or more neighbors share the same maximum number of connections, select the one that maximizes the likelihood function as compared to the other(s). If the candidate areas do not increase the likelihood, choose the one that least decreases the likelihood.

**Step 5**. Repeat steps 2 to 4 until the cluster reaches the maximum predetermined size.

**Step 6**. Repeat steps 1 to 5, until each area has been used as the start of the cluster.

**Step 7**. Note the cluster with the maximum likelihood ratio, obtained over all iterations in steps 1 to 6.

### Creating cylindrical cluster candidates with irregular base using mlink

**Figure 15:**
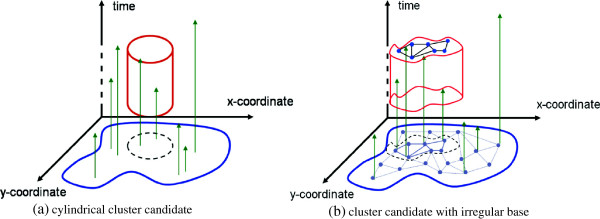
**Comparison of the standard space-time scan statistic and our first proposal.** The cylindrical cluster candidate varies the center and the radius of the base. We propose a cluster growing algorithm in which the base of the cylinder is created using the *mlink* algorithm.

Our first proposal of an irregular space-time permutation scan statistic is to use the *mlink* algorithm to build cylindrical candidates in which the base of the cylinder is irregular. Figure 15 illustrates the growing process of the cluster candidate. Suppose the height of the cylinder is fixed. Then, the standard cylindrical cluster algorithm varies the center and radius of the base, which is a circle. This is shown in Figure 15a. Our first *mlink* proposal, hereafter named *mlink-space*, creates cylinders with irregular base by applying the *mlink* algorithm to the base of the cylinder as shown in Figure 15b. The base of the cluster candidate is created applying the *mlink* algorithm, which requires the graph structure generated from spatial adjacency.

### Creating cluster candidates irregular in space and time using mlink

**Figure 16:**
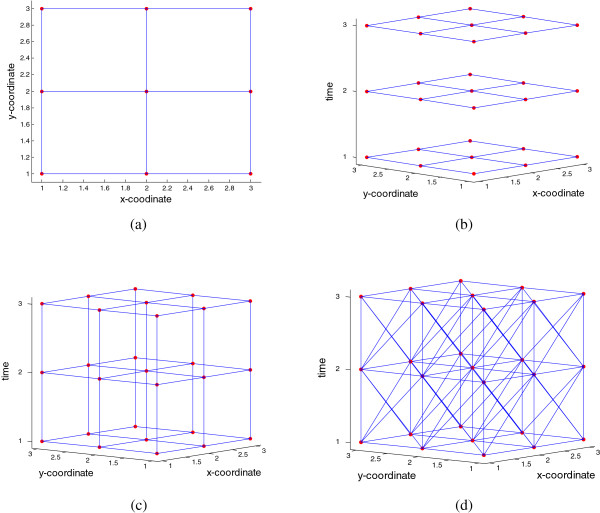
**Creating the space-time graph structure.** The algorithm starts from a regular spatial grid **(a)** which is replicated for each time **(b)**. The vertices are connected among the successive layers **(c)**, and finally, the vertices in each layer are connected to their respective neighbors in successive layers **(d)**.

Our second proposal, hereafter named *mlink space-time* creates cluster candidates which are irregular in space and time using the *mlink* algorithm. The process of growing cluster candidates is shown in Figure 16. Figure 16a illustrates a regular grid of 9 areas and the adjacency graph structure. First, we assume that the adjacency structure does not change in time. We do so because the space-time permutation procedure assumes that the spatial marginal distribution does not change in time under the null hypothesis. Therefore, for different times we have the same graph structure as shown in Figure 16b. Each vertex, or node shown in Figure 16b represents one area *i* of the studied regions at time *t* and the vertices are the adjacency structure. Second, we connect the graph structures. That is, each area *i* at time *t* is connected to its corresponding area *i* at times: *t* + 1 and time *t* - 1, as shown in Figure 16c. Finally, each area *i* at time *t* is also connected to its adjacent neighbors at times: *t* + 1 and time *t* - 1, as shown in Figure 16d. That is, neighbors in space are also neighbors in adjacent times. The final graph structure is composed of *K* × *T* edges in which the edges are the areas of the studied region for the different times, and the vertices represents a set of spatial and space-time adjacency structures. Furthermore, the final graph structure can be easily scanned with the standard *mlink* algorithm, since both temporal and spatial components no longer exist. Constraints on the temporal height of the cluster are set by allowing the cluster to aggregate only nodes which are within the pre-specified range of time. Alternatively, the growing process stops if cluster candidates reach the maximum number of nodes, previously defined by the user.

### Monte Carlo inference

After detecting the most likely cluster, it is important to evaluate how significant the candidate cluster is as compared to the null hypothesis of no space-time cluster. To do so, a set of replications of the original dataset is created under the null hypothesis randomization or permutation procedures. For each instance, the most likely cluster is found and its test statistic is accumulated generating an empirical distribution. Inference is carried out by estimating the p-value as the ratio of the number of simulations in which the simulated statistic is higher than the observed one over the total number of simulations plus one. This Monte Carlo procedure implies that the cluster search procedure is executed as many times as the resolution required for the p-value. Therefore, one of the possibilities for optimizing computing time includes decreasing the number of Monte Carlo simulations. However, if the number of simulations is reduced, then inference precision is compromised. Nevertheless, statistical procedures such as sequential Monte Carlo [21-23] can replace the conventional method and decrease the number of simulations.

### Sequential Monte Carlo P-values

The main drawback of the proposed algorithms is the computational cost. At each step, the algorithm must select a subset of the most connected areas and evaluate the likelihood statistic for each one of them. In addition, a Monte Carlo simulation procedure tests the null hypothesis of the random occurrence of the cluster, further increasing the time required for this data analysis. In general, the more arbitrary is the cluster search geometry and the larger are the spatial and temporal components, the more intense is the computing. In particular, for disease outbreak detection and daily surveillance the space-time permutation scan statistic is commonly applied on a daily basis. In some cases, multiple data streams analysis is required. Therefore, the computational burden is a critical component in the design of a daily surveillance system that promptly detects clusters with arbitrary shapes.

To efficiently reduce computational costs, we apply sequential Monte Carlo which reduces the expected number of Monte Carlo simulations under the null hypothesis. Briefly, the method searches for evidence that the null hypothesis is true from a smaller set of simulations. If it is found to be true, then the simulations are stopped; otherwise, the simulations are carried out using the conventional Monte Carlo analysis.

Sequential Monte Carlo sampling [21-23] optimizes computational costs assuming that the maximum number of simulations is a random variable. Let *U* defines the test statistic; *u* define the observed value; and *F* define its null distribution. The conventional Monte Carlo compares the value of *u* to the largest *n* random samples generated from *F*. If *u* is the *r-*th largest value from a total of *n* values, then the p-value is estimated as (*r + 1*)*/*(*n + 1*), which represents the significance level for the rejection of the null hypothesis. However, if there is evidence that the null hypothesis will not be rejected from a small sample of simulations, then the generation of large samples from the null hypothesis is both effortless and computationally expensive. In this situation, it is imperative to stop simulations as early as possible.

The sequential Monte Carlo produces exact p-values under the null hypothesis. The simulations are interrupted if *h* simulated values greater than the observed *u* are obtained; otherwise, it produces a maximum number of simulations (*n*). Let *l* be the number of Monte Carlo simulations in which *h* simulated values greater than *u* are obtained, *l* < *n*, the p-value is estimated as:

$$p_{s}=\left\{\begin{array}{cc} h/l, & \mathrm{if}\;g=h\\ \left(g+1\right)/(n+1), & \mathrm{if}\;g<h \end{array}\right)$$

where *g* is the number of simulated values greater than *u*, and *h* is the sequential parameter, Silva, Assunção and Costa [21] proved that for *h = 50*, there is no loss in power when compared to the exact test. Furthermore, in this situation, the expected number of simulations under the null hypothesis is equal to 115 [22].

## Competing interests

The authors declare that they have no competing interests.

## Authors’ contributions

MAC led the effort to draft the manuscript. MK guided the manuscript's development. MAC and MK read and approved the final manuscript.
